# Q Fever in Greenland

**DOI:** 10.3201/eid1603.091220

**Published:** 2010-03

**Authors:** Anders Koch, Claus Bo Svendsen, Jens Jørgen Christensen, Henning Bundgaard, Lars Vindfeld, Claus Bohn Christiansen, Michael Kemp, Steen Villumsen

**Affiliations:** University Hospital Rigshospitalet, Copenhagen, Denmark (A. Koch, H. Bundgaard, C.B. Christiansen); Statens Serum Institut, Copenhagen (A. Koch, C.B. Svendsen, J.J. Christensen, M. Kemp, S. Villumsen); Tasiilaq Health Center, Tasiilaq, Greenland (L. Vindfeld)

**Keywords:** Q fever, Coxiella burnetii, endocarditis, bacteria, Greenland, Arctic, Inuit, dispatch

## Abstract

We report a patient with Q fever endocarditis in a settlement in eastern Greenland (Isortoq, Ammassalik area). Likely animal sources include sled dogs and seals. Q fever may be underdiagnosed in Arctic areas but may also represent an emerging infection.

Q fever is a zoonosis caused by the small intracellular bacterium *Coxiella burnetii*. Main reservoirs for this bacterium are cattle, goats, and sheep, although a wide range of animals may be infected (*1**,**2*). *C*. *burnetii* can survive in a spore-like form under harsh conditions (*2*).

In animals, *C*. *burnetii* infection is often latent; the bacteria may be persistently shed into the environment, especially at the time of giving birth (*2*). In humans, most acute cases result in asymptomatic or mild influenza-like disease; severe disease develops in a few patients. Primary manifestations include pneumonia, hepatitis, and fever of unknown origin.

Q fever has been described in >59 countries (*1*) but not in Arctic areas. We report a patient with Q fever in Greenland.

## The Patient

The patient, a 40-year-old man, who resided in Greenland all his life, lived in Isortoq (population 100), a small settlement in the Ammassalik area (population 3,000) of eastern Greenland (Figure). He had worked as a hunter and a sanitation worker (garbage collector). The Ammassalik area includes the main town of Tasiilaq and 5 settlements. Isortoq is located on an island off the coast of Greenland. Access is by helicopter, boat during the summer, and dog sleds and snowmobiles during the winter. The main occupation is hunting, especially of seals, which are consumed locally. All other food is imported though Tasiilaq. All imported meat is frozen, and only ultra-high-temperature–pasteurized milk is available. Terrestrial mammals in the area include sled dogs, polar foxes, and a few domesticated cats. Sea mammals include seals and walruses. Polar bears are abundant throughout eastern Greenland; the nearest sheep, horses, and musk oxen are >1,000 km away. There are no cows and goats in Greenland.

**Figure:**
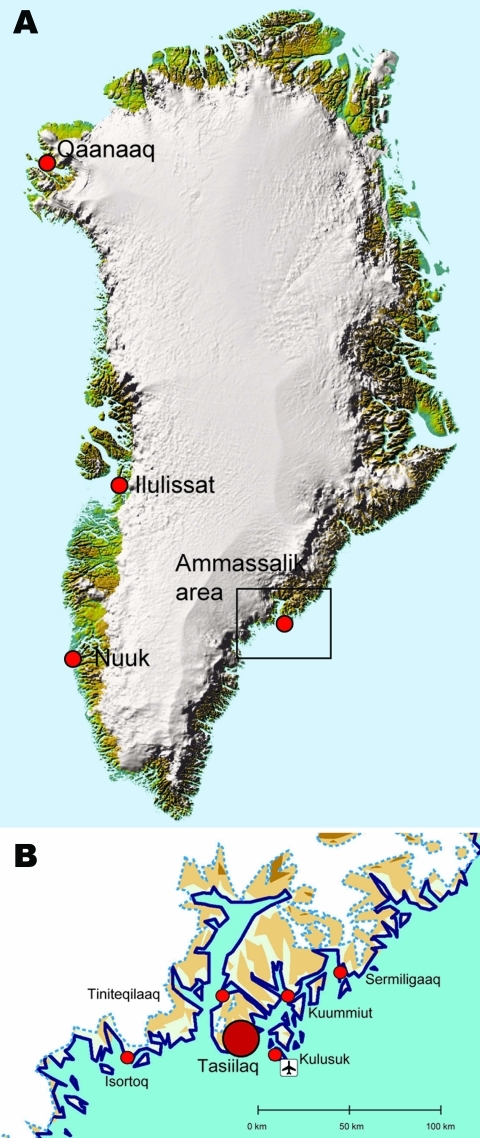
A) Ammassalik area (box) in Greenland. B) Main towns in the Ammassalik area. Red circles show the main town of Tasiilaq and 5 settlements. Location of the airport is indicated. Reprinted with permission of the National Survey and Cadastre [Kort og Matrikelstyrelsen], Danish Ministry of the Environment, Copenhagen, Denmark.

In December 2007, the patient came to Tasiilaq Health Center with dyspnea, chest pains, and fever that had lasted 2 months. In 2001, because he had had rheumatic fever during childhood, he had biological aortic and mitral valves implanted. As a result, he was at increased risk for Q fever endocarditis (*3*). However, he was healthy and had not taken any medications during the preoperative period.

In January 2008, the patient was transferred to University Hospital Rigshospitalet in Copenhagen, Denmark. Clinical findings included low-grade fever, cardiac insufficiency with peripheral edema, hepatosplenomegaly, and 20% half-moon nephritis; a transesophageal echocardiograph did not show signs of endocarditis. Repeated blood cultures were negative for bacteria.

In May 2008, an echocardiograph showed aorta and mitral valve vegetations and stenoses. Subsequent surgery showed massive endocarditis. His biological valves were replaced with mechanical valves. Recovery was uneventful, signs of heart failure disappeared, and laboratory test results and cardiac function gradually returned to reference levels.

A sample from his resected cardiac valves was subjected to routine partial 16S rRNA PCR and DNA sequencing (*4*). A BLAST search in the National Center for Biotechnology Information (Bethesda, MD, USA) database showed 502 of 502 bp to be identical with those of *C*. *burnetii*. Identification was confirmed by PCR specific for the *C*. *burnetii* transposase (IS*1111*a) gene by using primers CoxiellaF1x (5′-GTA TCG GAC GTT TAT GGG GAT GGG TAT CC-3′) and CoxiellaR1 (5′-CAC CAC GCG CCA TCG TGA GTC-3′). PCR conditions were 10 cycles at 95°C for 30 s and 75°C–65°C for 60 s and 40 cycles at 95°C for 30 s and 65°C for 60 s.

Subsequent culture in Vero cells was positive after incubation for 30 days, and results were confirmed by indirect immunofluorescent assay with *C*. *burnetii* phase II–specific antibodies (Australian Rickettsial Reference Laboratory, Geelong, Victoria, Australia). A nearly full-length sequence of the 16S rRNA gene was obtained by DNA sequencing of culture material and yielded 1,321- of 1,321-bp sequence homology with *C*. *burnetii*, including the original sequence obtained directly from the valve. This DNA sequence has been submitted to GenBank (accession no. 1188993 FJ787329) as the Ammassalik strain.

Blood obtained 27 days before surgery was positive by *C*. *burnetii*–specific PCR. A sample contained high levels of *C*. *burnetii*–specific antibodies by immunofluorescent assay (Focus Diagnostics, Cypress, CA, USA): immunoglobulin (Ig) M titer phase I, 16,000; IgM phase II, 16,000; IgG phase I, 512,000; and IgG phase II, 1,024,000.

In April 2004, the patient had participated in a population-based screening in the Ammassalik area for antibodies against *Trichinella* spp. and *Anisachis* spp. (*5*). His serum was stored at –80°C at Statens Serum Institut and tested for antibodies against *C*. *burnetii* after illness was reported in 2008; results for IgM and IgG were negative. Except for his cardiac surgery in 2001 in Copenhagen, the patient had never been outside the Ammassalik area.

## Conclusions

The patient was likely infected in the Ammassalik area in 2007, rather than during or before 2004. His stored serum sample was negative for *C*. *burnetii* in 2004. The lack of domesticated ruminants may be the reason why Q fever has not been described in Arctic areas. Although *C*. *burnetii* has not been isolated from Arctic animals, some musk oxen in northern Quebec and reindeer in Arctic Russia (Nenet region) have been found to be positive for IgG against *C*. *burnetii* (*6**,**7*). Likewise, <0.6% of Inuits from Nunavik, 15% of trappers, and 18% of Cree hunters from interior regions of southern Quebec have been found to be positive for IgG against *C*. *burnetii* (*8**–**10*).

In the absence of raw milk products, animals represent the most likely source of infection in eastern Greenland. *C*. *burnetii* has been found in dogs, cats, birds (*2*), seals (*11*), and bears (*12**,**13*) in other regions. For our patient, we cannot rule out the possibility that infection may have been caused by a domestic cat that may have traveled with its owner to a region endemic for Q fever or by migratory birds. However, because endocarditis is a rare manifestation of Q fever and affects <0.5% of all case-patients (*2*), *C*. *burnetii* may be endemic to the Arctic area. The most likely animal reservoirs would be sled dogs or seals because a herd of a certain size is necessary to sustain infection in an animal population. Sled dogs are mostly kept chained in groups, and bacteria may spread from infected placentas to other dogs and humans in the vicinity. Seals are abundant in the Ammassalik area and represent a major human food source. Harbor and hooded seals form colonies at time of giving birth, when infection is most likely to spread (*11*). Polar foxes and bears are less likely reservoirs because their populations are less dense (*2*).

Whether Q fever is an underdiagnosed or emerging disease in the Arctic area is unknown. Because many cases are asymptomatic and laboratory facilities in Greenland are few, this disease, although rare, may be underdiagnosed in this country. However, Q fever may also be an emerging infection, possibly related to climate changes, as seen elsewhere in the Arctic area (*14*).

Serologic studies of persons in the Arctic region, including Greenland, may shed light on these issues. Possible animal reservoirs may be identified by serologic studies of wildlife. In addition, health authorities in the Arctic area need to be aware of *C*. *burnetii* as a possible infectious agent.
